# Measuring stability and change in response patterns to a set of hierarchical scales in a randomized intervention study: an innovative application of latent transition analysis

**DOI:** 10.1007/s10865-025-00586-7

**Published:** 2025-08-25

**Authors:** Nicholas D. Myers, Isaac Prilleltensky, Seungmin Lee

**Affiliations:** 1 Department of Kinesiology, Michigan State University, 201 IM Sports Circle Building, 308 W. Circle Drive, East Lansing, MI 48824 USA; 2 Department of Educational and Psychological Studies, University of Miami, Coral Gables, FL USA; 3 Department of Kinesiology, Iowa State University, Ames, IA USA

**Keywords:** Latent class, Physical activity promotion, e-Health, m-Health, Physical activity self-efficacy

## Abstract

The objective of this study was to measure stability and change in response patterns to a set of hierarchical Physical Activity Self-Efficacy (PASE) Scales with latent transition analysis (LTA). To accomplish this objective a multiple-group LTA modeled binary responses to six ordered items within each PASE scale. Data (*N*_*baseline*_ = 461 and *N*_*30 days post-baseline*_ = 428) from the Well-Being and Physical Activity (WBPA; ClinicalTrials.gov, identifier: NCT03194854) study were analyzed. A four-class solution with interpretable parameter restrictions explained response patterns to each PASE scale at baseline. Evidence for temporal measurement invariance of this four-class solution was provided. Stability of latent class membership from baseline (i.e., pre-intervention) to 30 days post-baseline (i.e., post-intervention) was modest, consistent with substantive theory. Desirable differences in LTA probabilities (e.g., transitioning from confidence to engage in 10 min of weekly physical activity at baseline to confidence to engage in 90 min of weekly physical activity at 30 days post-baseline) by intervention group were observed, consistent with objectives of the WBPA study.

## Introduction

Mixture modeling permits heterogeneity in data, where observations can belong to various subpopulations without directly observing subpopulation membership, via categorical latent variables (e.g., Everitt & Hand, 1981; Goodman, 1974; Lazarsfeld, 1950; McLachlan & Basford, 1988; Muthén, 1989; Muthén & Shedden, 1999; Titterington et al., 1985). Latent transition analysis (LTA) is a mixture model (e.g., Bye & Schechter, 1986; Langeheine & Rost, 1988; Mooijaart, 1998) that typically takes an auto-regressive approach (i.e., regressing subsequent latent classes on previous latent classes repeatedly measured by a set of categorical indicators) to describe stability and change in categorical states (i.e., classes) across time via transition probabilities (e.g., Collins & Lanza, 2010; Hagenaars & McCutcheon, 2002). These estimated transition probabilities typically are focal parameters and distinguish LTA from other more commonly implemented latent change models (e.g., Rogosa & Willett, 1985).

While applications of LTA have recently increased (e.g., Nylund-Gibson et al., 2023), multiple-group LTA where the grouping variable is randomization to an intervention within a randomized controlled trial (RCT) is relatively rare (e.g., Lanza & Rhoades, 2013). Multiple-group LTA where the observed grouping variable is randomization to an intervention within a RCT has the potential to answer an important research question: do transition probabilities for latent classes of a theory-driven malleable intervention target differ by intervention groups? Answering this question has the potential to identify for whom (e.g., latent subpopulations) an intervention may be effective. Knowing for whom an intervention is likely effective has implications for optimizing delivery of an intervention (e.g., Collins & Kugler, 2018). Focusing on “for whom” research questions may be well-served by person-centered models (e.g., mixture models), where key data may be person-level (e.g., response patterns to a set of categorical indicators) instead of (or in addition to) a more common focus on variable-level (e.g., covariance matrix and/or mean vector for a set of continuous indicators) data (e.g., Morin & Litalien, 2019). For whom research questions often are exploratory aims of an RCT that are investigated in secondary analyses after evidence for variable-centered models investigating primary variable-level research questions has been provided (e.g., Collins & Lanza, 2010; Howe & Leitjen, 2023).

### The well-being and physical activity (WBPA) study

The objective of the 2018 WBPA study (Myers et al., 2019) was to provide the first investigation of the effectiveness of the Fun For Wellness (FFW; Myers et al., 2017) intervention to increase well-being and physical activity in adults with obesity in the United States of America (USA). There were three main outcomes in the WBPA study: subjective well-being, well-being actions, and physical activity. A separate conceptual model was constructed for each of these outcomes; that is, the FFW conceptual model for the promotion of: (1) subjective well-being (Myers et al., 2021a, 2021b); (2) well-being actions (Lee et al., 2021); and, (3) physical activity (Myers et al., 2020). Within each of the FFW conceptual models a self-efficacy construct (e.g., well-being self-efficacy; well-being actions self-efficacy; and, physical activity self-efficacy) was targeted as a theory-driven malleable mediator of a concordant main outcome (i.e., subjective well-being; well-being actions; and physical activity, respectively). Data collection details relevant to the current study will be reported in the Method section. Readers are referred to: Lee et al. (2021) for well-being actions results; and, Myers et al. (2021a, 2021b) and Myers et al. (2023a, 2023b) for subjective well-being results. Given that the focus of the current manuscript is on physical activity self-efficacy, results under a variable-centered approach reported in Myers et al. (2021a, 2021b) will be reviewed below. Before doing so, however, we provide a brief review of the population, behavior change theory, intervention, and measurement of physical activity self-efficacy in the WBPA study.

#### Population

Obesity is a risk factor for major non-communicable chronic diseases such as cardiovascular disease, type II diabetes, musculoskeletal disorders, and some cancers (United States Department of Health and Human Services [USDHHS], 2013). Billions of adults are with overweight, and either are, or are at risk for, obesity (World Health Organization [WHO], 2018). Specifically, approximately one-third of adults with overweight (i.e., 25.00 kg/m^2^ ≤ body mass index [BMI]) can more precisely be classified as adults with obesity (i.e., BMI ≥ 30.00 kg/m^2^). The magnitude of observations within the obese category has increased threefold over the past few decades (WHO, 2018). To reduce the prevalence of adults with obesity, the WHO () and the USDHHS (2018) both recommend that individuals engage in regular physical activity. One way to meet these recommendations is to engage in at least 150 min of physical activity at a moderate (which many adults with obesity prefer over vigorous) intensity each week (e.g., Baillot et al., 2021). There is evidence, however, that a large percentage of adults with obesity do not meet public health guidelines for physical activity (Tran et al., 2020; Tudor-Locke et al., 2010). Fortunately, there is also evidence that cognitive-behavioral interventions can promote physical activity in adults with obesity (e.g., Gourlan et al., 2011; Lee et al., 2023, 2025).

#### Behavior change theory

The behavior change theory used in the WBPA study was social cognitive theory (Bandura, 1986, 2001). Self-efficacy theory (Bandura, 1977, 1997) resides within social cognitive theory and views an individual as a proactive agent in the regulation of their emotions, cognitions, and behaviors. Self-efficacy beliefs play a primary role in self-efficacy theory and are defined as domain-specific judgments held by an individual about their ability to successfully execute differing levels of performance given certain situational demands. Self-efficacy theory posits that a “self-efficacy for varying levels of performance” construct may play a central role in the initiation of a behavior (e.g., engaging in physical activity for at least 10 min, 30 min, 60 min, etc. in the next week; Myers et al., 2020), whereas “self-efficacy to regulate a behavior” construct may play a central role in the maintenance of a behavior (e.g., continually engaging in a recommended amount of weekly physical activity even when the weather is bad; McAuley, 1993). The focus of the current study is measuring stability and change in physical activity self-efficacy level (not self-regulation) because many adults with obesity do not continually engage in physical activity at a level that meets public health guidelines.

Self-efficacy beliefs rely on the cognitive processing of several potential sources of efficacy information: enactive mastery experiences, vicarious experiences, verbal persuasion, and physiological and/or emotional states. Two proposed omnibus outcomes of self-efficacy beliefs are an individual’s thought patterns (e.g., goal setting, worry, and attributions) and behaviors (e.g., challenges undertaken, effort expended on challenges undertaken, and persistence in the face of difficulties that arise during challenges undertaken). Two necessary conditions for valid testing of self-efficacy theory are: (a) high degree of concordance between proposed sources of self-efficacy information and domain specific self-efficacy beliefs and (b) a self-efficacy scale constructed to measure the unique nature and structure of domain specific self-efficacy beliefs (Bandura, 1977, 1997, 2006). Meeting both of these conditions was necessary to measure stability and change in physical activity self-efficacy in the WBPA study.

#### The intervention

Fun For Wellness is a self-efficacy theory-based online behavioral intervention developed to promote growth in well-being and physical activity by providing capability-enhancing opportunities to participants (Myers et al., 2019). Capability-enhancing opportunities provided to FFW participants come in the form of 152 interactive and scenario-based challenges organized in the on-line environment by the BET I CAN acronym (Myers et al., 2017). The capability-enhancing learning opportunity within each of the 152 BET I CAN challenges provides a FFW participant with an exposure to one or more of Bandura’s potential sources of self-efficacy information (Myers et al., 2017). More specifically, each BET I CAN challenge provides an opportunity for a participant to engage in one of the following activities: (a) playing an interactive game (i.e., exposure to an enactive mastery experience), (b) watching a vignette performed by professional actors (i.e., exposure to a vicarious experience), (c) listening and/or reading a mini-lecture narrated by a coach (i.e., exposure to a verbal persuasion experience), and (d) engaging in a self-reflection exercise and/or a chat room (i.e., exposure to an assessment of relevant physiological and/or emotional states experience). A central tenant in the FFW conceptual model for the promotion of physical activity was that exposure to the BET I CAN challenges would promote physical activity self-efficacy.

#### Measurement of physical activity self-efficacy

Four hierarchical physical activity self-efficacy (PASE) Scales for moderate intensity physical activity were developed for the WBPA study consistent with guidelines for constructing self-efficacy scales in general (e.g., Bandura, 1997, 2006) and physical activity domain specificity in particular (e.g., Feltz & Chase, 1998). Specific physical activity domains (i.e., work-, transport-, domestic-, and leisure-related) were specified across the battery of PASE scales to be concordant with how physical activity was measured in the WBPA study (i.e., with the International Physical Activity QuestionnaireAinsworth et al., 2000; Craig et al., 2003). *Work-related moderate intensity physical activity self-efficacy* was defined as the degree to which an individual perceives that they have the capability to engage in job-related physical activity at a moderate intensity in the next week. *Transport-related moderate intensity physical activity self-efficacy* was defined as the degree to which an individual perceives that they have the capability to engage in transport-related physical activity at a moderate intensity in the next week. *Domestic-related moderate intensity physical activity self-efficacy* was defined as the degree to which an individual perceives that they have the capability to engage in domestic-related physical activity at a moderate intensity in the next week. *Leisure-related moderate intensity physical activity self-efficacy* was defined as the degree to which an individual perceives that they have the capability to engage in leisure-related physical activity at a moderate intensity in the next week. The ordered gradations of challenge in these PASE Scales ranged from a minimum (i.e., *for at least 10 min in the next week*) to a maximum (i.e., *for at least 150 min in the next week*) typically in 30-min increases (i.e., 10, 30, 60, 90, 120, and 150). The hierarchical structure in the PASE scales is likely to present methodological challenges when estimating mixture models (e.g., Rindskopf, 1983) that will be detailed in the Method section.

Responses to a PASE Scale can be modeled to measure a physical activity self-efficacy *level* or *strength* construct (e.g., Myers et al., ). A physical activity self-efficacy *strength* construct measures the degree of certainty of a person’s belief (e.g., 0 = no, 1 = low, 2 = moderate, 3 = high, and 4 = complete confidence) that they can attain a given gradation of challenge (e.g., engaging in physical activity for at least 10 min in the next week). Measuring a self-efficacy *strength* construct requires an ordinal indicator format because the conceptual focus of the construct is the degree of certainty of a person’s belief (e.g., Bandura, 1977, 1997, 2006; Feltz et al., 1998). A physical activity self-efficacy *level* construct measures the highest gradation of challenge (e.g., engaging in physical activity for at least 10 min in the next week) a person believes they may be able obtain (e.g., 0 = no, 1 through 4 = yes). For example, a person who responds with moderate confidence (e.g., 2) that they can engage in physical activity for at least 10 min in the next week, and then responds with no confidence (e.g., 0) that they can engage in physical activity for at least 30 min in the next week, would have a level of at least 10 min for the next week. Measuring a self-efficacy *level* construct requires a binary (versus ordinal) indicator format because the conceptual focus of the construct is binary: a level is not passed versus a level is passed (e.g., Bandura, 1977, 1997, 2006; Feltz et al., 1998). Myers et al. (2021a, 2021b) modeled responses to each PASE Scale under a traditional variable-centered approach (i.e., the common factor model with a single class) to measure physical activity self-efficacy strength consistent with the dominant approach in physical activity intervention research for adults with obesity (e.g., Bateman et al., 2022).

#### Results under a traditional variable-centered approach

An important component of construct validity in educational and psychological testing is the internal structure of a test: “The degree to which the relationships among test items and test components conform to the construct on which the proposed test score interpretations are based” (American Educational Research Association [AERA] et al., 2014, p.14). Evidence for a two-dimensional internal structure of responses to the PASE Scales in the WBPA study under a variable-centered approach to measure physical activity self-efficacy strength was provided in Myers et al. (2021a, 2021b)*.* Latent construct reliability estimated with coefficient *H* (Hancock & Mueller, 2001) ranged from 0.97 to 0.99 for an *up to 60 min factor* and was 0.99 for a *60 min or more factor* across the PASE Scales. This two-factor exploratory structural equation model (Asparouhov & Muthén, 2009) passed the exact fit test in each PASE Scale. The internal structure of responses to the PASE Scales under mixture modeling has yet to be examined.

Another important component of construct validity in educational and psychological testing is the degree to which responses to an instrument relate to variables external to the instrument consistent with substantive theory (AERA et al., 2014). A self-efficacy theory-based hypothesis tested in Myers et al. (2021a, 2021b) was that assignment to the FFW intervention would exert a positive direct effect on latent physical activity self-efficacy strength at 30-days post-baseline. There was uniform evidence for the effectiveness of the FFW intervention to exert a positive direct effect on work-, transport-, domestic-, and leisure-related latent physical activity self-efficacy strength for the shorter duration (i.e., up to 60 min factor) but not the longer duration (i.e., 60 min or more factor). This variable-centered approach yielded important insights but did not address for whom (e.g., latent subpopulations) the FFW intervention may be effective in promoting physical activity self-efficacy.

### Objective and exploratory research questions

The objective of this study was to measure stability and change in person-level response patterns to the PASE Scales in the WBPA study with LTA. To accomplish this objective a multiple-group LTA modeled binary responses to six ordered items within each PASE Scale to investigate four exploratory research questions.

*Research Question 1.* How many latent classes (i.e., class enumeration) were warranted to explain response patterns to each PASE Scale?

*Research Question 2.* Was there evidence for temporal measurement invariance of the accepted latent class analysis (LCA) solution for each PASE Scale?

*Research Question 3.* What were the unconditional transition probabilities (i.e., stability and change) for latent classes prior to intervention to post-intervention in each PASE Scale?

*Research Question 4.* Did the transition probabilities for latent classes differ by intervention group in each PASE Scale?

To investigate these questions a multiple study design was used because results from a former research question had implications for the investigation of subsequent research questions. For example, results from investigating class enumeration (i.e., Research Question 1) had implications for model specification investigating temporal invariance (i.e., Research Question 2). Consistent with a multiple study design, a general method section is first provided followed by study-specific method, results, and conclusion sections prior to a brief general discussion.

## General method

The data described in this manuscript were collected in the WBPA study (ClinicalTrials.gov, identifier: NCT03194854). The PASE Scale data analyzed in this manuscript were analyzed by Myers et al. (2021a, 2021b) under a variable-centered approach with the common factor model to measure physical activity self-efficacy *strength.* These data were reanalyzed under a person-centered approach with mixture modeling in the current manuscript to investigate four new exploratory research questions regarding physical activity self-efficacy *level*. Investigating these four new exploratory research questions was important because (a) self-efficacy theory posits that a “self-efficacy for varying levels of performance” construct (e.g., Bandura, 1977, 1997, 2006) may play a central role in the initiation of a behavior (e.g., engaging in physical activity for at least 10 min, 30 min, 60 min, etc. in the next week; Myers et al., 2020) and (b) many adults with obesity have yet to initiate continual engagement in physical activity at a level that meets public health guidelines (Tran et al., 2020; Tudor-Locke et al., 2010). Some of the text that describe the methods used in the WBPA study is similar to text in Myers, Bateman, et al. We provide this text so that the reader does not need to consult a previously published paper to understand methods used in the WBPA study that are important to understand the data used in the present study (American Psychological Association, 2020).

### Research design

Recruiting, screening, random assignment and data collection were conducted online from August 2018 through November 2018. Data collection occurred at three time points: baseline, 30 days, and 60 days post-baseline. This timeline was similar to timelines used in other physical activity-promoting interventions in adults with obesity (USPSTF, 2018). Data collected at baseline and 30 days post-baseline were of interest in the current study based on the FFW conceptual model for the promotion of physical activity (Myers et al., 2020), which posited that exposure to the FFW intervention from baseline to 30 days post-baseline would increase physical activity self-efficacy at 30 days post-baseline.

### Eligibility and recruitment

Eligibility criteria were: (a) ability to access the online intervention; (b) living in the USA; (c) 18 years old ≤ age ≤ 64 years old; (d) BMI ≥ 25.00 kg/m^2^; and, (e) absence of simultaneous enrollment in another intervention program promoting well-being and/or physical activity. The BMI criterion included both overweight and obese categories consistent with many physical activity-promoting interventions for adults with obesity (e.g., Gourlan et al., 2011; Lee et al., 2023, 2025). Participants were recruited across the USA via the general population panel of the SurveyHealth recruitment company consistent with a worldwide movement toward larger and smarter physical activity-promoting interventions (e.g., Reis et al., 2016).

### Random assignment

Random assignment of each eligible participant occurred after a unique and secure login credential was created, informed consent was obtained, a medical disclaimer was agreed to, and the baseline survey battery was completed. Eligible participants were randomly assigned to the intervention (i.e., FFW) or the usual care (i.e., UC) group via software code that was written to accomplish approximately equal allocations to the two groups. Participants assigned to the FFW group were given immediate 30-day access to the intervention. Participants assigned to the UC group were put on a waitlist for access to the intervention at the conclusion of the WBPA study.

### PASE scales

Four hierarchical PASE Scales designed to measure physical activity self-efficacy for moderate intensity physical activity—Work-Related Moderate Intensity (WRMI), Transport-Related Moderate Intensity (TRMI), Domestic-Related Moderate Intensity (DRMI), and Leisure-Related Moderate Intensity (LRMI)—were included in the survey battery. The battery of PASE scales was developed within the context of the FFW intervention (i.e., an unstructured physical activity-promoting program) and assessed the degree to which an individual perceives that they have the capability to engage in a recommended amount of weekly physical activity for health. A recommended amount of weekly physical activity for health was defined to be concordant with both the WHO () and USDHHS (2013, 2018). The six ordered gradations of challenge (i.e., items) in these PASE Scales ranged from a minimum (i.e., *for at least 10 min in the next week*) to a maximum (i.e., *for at least 150 min in the next week*) typically in 30-min increases (i.e., 10, 30, 60, 90, 120, and 150). See Appendices A-D in the supplemental materials for a copy of each PASE Scale for further details.

### Data collection, demographics, and descriptive statistics

Readers are referred to Myers et al. (2020) for a figure depicting participant flow from screening to randomization to retention over the measurement occasions. In summary, 461 (*n*_FFW_ = 219, *n*_UC_ = 242) and 427 or 428 participants (*n*_FFW_ = 197 or 198, *n*_UC_ = 230) provided PASE Scale data at baseline and 30 days post-baseline, respectively. Sample size at 30 days post-baseline was 428 for responses to the WRMI PASE Scale and 427 for all other PASE Scales because one participant did not respond to any item within the latter three PASE scales. Beyond attrition (i.e., 7.4% of participants from baseline did not provide PASE Scale data at 30 days post-baseline) there were no missing data on variables used in this manuscript. A majority of the participants identified as female (65.5%), White, non-Hispanic (74.2%), having completed at least a 4-year college degree (66.5%), married (68.2%), a full-time employee (67.0%), at least 40-years old (55.1%), and as residing in a household with an annual income of at least $75,000 (51.6%). There were no statistically significant differences in the observed proportions of demographic characteristics or the items in each PASE Scale at baseline by randomization group. A majority (91.7%) of the participants who were assigned to the FFW group were classified as engaged with the FFW intervention (see Lee et al., 2024, and Scarpa et al., 2021 for full engagement results).

Six binary indicators were created for each PASE Scale by leaving the 0 category intact (i.e., “no” confidence) and collapsing responses greater than 0 (i.e., 1,2,3,4) to 1 (i.e., at least low confidence or “yes”) consistent with the measurement of a self-efficacy level construct (e.g., Bandura, 1997, 2006; Feltz & Chase, 1998). Recall that a self-efficacy level construct communicates the final gradation of challenge (i.e., an ordered item) at which at least some confidence is reported by a participant. Thus, the final “yes” observed for each participant was their observed physical activity self-efficacy level. Note that a “no” response at the first gradation of challenge (i.e., 10 min) meant that a physical activity self-efficacy level was not observed (i.e., less than 10 min) for a participant. Similarly, a “yes” response to each of the six ordered gradations of challenge meant that a physical activity self-efficacy level may not have been observed (i.e., at or more than 150 min) for a participant. These boundary cases (i.e., floor—less than 10 min; ceiling—at or more than 150 min) are common and of substantive interest in the measurement of self-efficacy level because they represent extreme sub-populations.

Table 1 provides the frequency distributions for the six binary items within each PASE Scale item by response category at baseline and 30 days post-baseline. The same general pattern of responses was observed in each PASE Scale at both time points. Specifically, observations in the “no” category increased monotonically from the first item (i.e., 10 min) to the sixth item (i.e., 150 min) within each PASE Scale at both time points. For example, at baseline in the WRMI PASE Scale, 20.0% of observations were observed in the “no” category for the first item (i.e., 10 min) while 69.8% of observations were observed in this category for the sixth item (i.e., 150 min). Conversely, observations in the “yes” category necessarily decreased monotonically from the first item (i.e., 10 min) to the sixth item (i.e., 150 min) within each PASE Scale. For example, at baseline in the WRMI PASE Scale, 80.0% of observations were observed in the “yes” category for the first item (i.e., 10 min) while 30.2% of observations were observed in this category for the sixth item (i.e., 150 min).

**Table 1:** Frequency distributions for six binary items within each physical activity self-efficacy (PASE) scale at baseline and 30 days post-baseline

PASE Scale	Item	Baseline (*N* = 461)		30 Days Post-Baseline (*N* = 427)	
		No (%)	Yes (%)	No (%)	Yes (%)
Work-related moderate intensity (WRMI)^a^	For at least 10 min in the next week	20.0	80.0	20.1	79.9
	For at least 30 min in the next week	33.8	66.2	32.5	67.5
	For at least 60 min in the next week	47.7	52.3	51.6	48.4
	For at least 90 min in the next week	59.9	40.1	66.8	33.2
	For at least 120 min in the next week	67.2	32.8	76.6	23.4
	For at least 150 min in the next week	69.8	30.2	79.0	21.0
Transport-related moderate intensity (TRMI)	For at least 10 min in the next week	14.8	85.2	19.4	80.6
	For at least 30 min in the next week	29.5	70.5	33.3	66.7
	For at least 60 min in the next week	45.3	54.7	53.2	46.8
	For at least 90 min in the next week	61.6	38.4	66.0	34.0
	For at least 120 min in the next week	70.3	29.7	76.8	23.2
	For at least 150 min in the next week	73.3	26.7	80.8	19.2
Domestic-related moderate intensity (DRMI)	For at least 10 min in the next week	10.0	90.0	11.2	88.8
	For at least 30 min in the next week	22.8	77.2	21.8	78.2
	For at least 60 min in the next week	38.8	61.2	46.8	53.2
	For at least 90 min in the next week	56.4	43.6	62.3	37.7
	For at least 120 min in the next week	65.7	34.3	73.3	26.7
	For at least 150 min in the next week	68.5	31.5	76.1	23.9
Leisure-related moderate intensity (LRMI)	For at least 10 min in the next week	10.4	89.6	12.6	87.4
	For at least 30 min in the next week	22.1	77.9	23.7	76.3
	For at least 60 min in the next week	38.2	61.8	43.8	56.2
	For at least 90 min in the next week	55.7	44.3	60.9	39.1
	For at least 120 min in the next week	64.6	35.4	72.6	27.4
	For at least 150 min in the next week	68.1	31.9	76.6	23.4

^a^*N* = 428 for the Work-related moderate intensity PASE Scale at 30 Days Post-Baseline

Table 2 provides observed response patterns to the six binary items within each PASE Scale at baseline and 30 days post-baseline. Note that only seven response patterns were possible due to the hierarchical nature of each PASE Scale and the fully automated web-based programming in the WBPA study. Specifically, once a participant selected 0 (i.e., “no” confidence) for an item (e.g., 30 min) they did not respond to remaining ordered items (i.e., 60, 90, 120, 150 min) and were scored 0 for these items within the relevant PASE Scale. Thus, disordered response patterns within each PASE Scale (e.g., 0,1,1,1,1,1) were not possible in the WBPA study. Each possible response pattern was given a name corresponding to the final level at which a 1 response was observed (e.g., 1,0,0,0,0,0 = 10 min). At least some responses were observed in each possible response pattern although the 120 min level (i.e., response pattern: 1,1,1,1,1,0) was rarely used (e.g., ≤ 4.0%) in each PASE Scale at both time points. The ceiling (i.e., ≥ 150 min) level (i.e., response pattern: 1,1,1,1,1,1) was the most frequently observed response pattern (i.e., ~ 30% of participants) in each PASE Scale at baseline. This highly efficacious response pattern was used less frequently at 30 days post-baseline (i.e., ~ 20% of participants), consistent with theoretical expectations (i.e., self-efficacy beliefs tend to become more realistic with increased attention to related experiences; Bandura, 1977, 1997).

**Table 2:** Response patterns to six binary items within each physical activity self-efficacy (PASE) scale at baseline and 30 days post-baseline

PASE Scale	Item Response Patterns^a^	Level	Baseline (*N* = 461) (%)	30 Days Post-Baseline (*N* = 427) (%)
Work-related moderate intensity (WRMI)^b^	0,0,0,0,0,0	< 10 min	20.0	20.1
	1,0,0,0,0,0	10 min	13.9	12.4
	1,1,0,0,0,0	30 min	13.9	19.2
	1,1,1,0,0,0	60 min	12.1	15.2
	1,1,1,1,0,0	90 min	7.4	9.8
	1,1,1,1,1,0	120 min	2.6	2.3
	1,1,1,1,1,1	≥ 150 min	30.2	21.0
Transport-related moderate intensity (TRMI)	0,0,0,0,0,0	< 10 min	14.8	19.4
	1,0,0,0,0,0	10 min	14.8	13.8
	1,1,0,0,0,0	30 min	15.8	19.9
	1,1,1,0,0,0	60 min	16.3	12.9
	1,1,1,1,0,0	90 min	8.7	10.7
	1,1,1,1,1,0	120 min	3.0	4.0
	1,1,1,1,1,1	≥ 150 min	26.7	19.2
Domestic-related moderate intensity (DRMI)	0,0,0,0,0,0	< 10 min	10.0	11.2
	1,0,0,0,0,0	10 min	12.8	10.5
	1,1,0,0,0,0	30 min	16.1	25.0
	1,1,1,0,0,0	60 min	17.6	15.4
	1,1,1,1,0,0	90 min	9.3	11.0
	1,1,1,1,1,0	120 min	2.8	2.8
	1,1,1,1,1,1	≥ 150 min	31.5	23.8
Leisure-related moderate intensity (LRMI)	0,0,0,0,0,0	< 10 min	10.4	12.6
	1,0,0,0,0,0	10 min	11.7	11.0
	1,1,0,0,0,0	30 min	16.1	20.1
	1,1,1,0,0,0	60 min	17.6	17.1
	1,1,1,1,0,0	90 min	8.9	11.7
	1,1,1,1,1,0	120 min	3.5	4.0
	1,1,1,1,1,1	≥ 150 min	31.9	23.4

^a^0 = No and 1 = Yes to a PASE Scale Item

^b^*N* = 428 for the Work-related moderate intensity PASE Scale at 30 Days Post-Baseline

### Data analytic approach

Mixture models were fit to responses to each PASE Scale separately in M*plus* 8.11 (Muthén & Muthén, 1998–2017). Maximum likelihood (ML) estimation for categorical variable methodology with a logit link function was used (Muthén, 1984). Type I error rate was set equal to 0.05. Missing data were addressed with full information ML estimation under the assumption of missing at random (Rubin, 1976) based on handling missing data in LCA (e.g., Kolb & Dayton, 1996). In each subsequent study, an exploratory approach was typically used consistent with the exploratory nature of this study.

## Study 1: class enumeration

### Method

How many latent classes were warranted to explain response patterns to each PASE Scale was first estimated via LCA at both time points independently (e.g., Nylund-Gibson et al., 2023). A necessary condition for LCA identification was that the number of parameters to be estimated (*P*) was less than the number of possible response patterns (*W*). In a typical unrestricted LCA with *M* number of binary indicators (i.e., *u*_1_, *u*_*2*_,…*u*_J_) of latent class variable *C* with *K* number of latent classes, *P* includes *K-1* latent class means (i.e., **a**; structural parameters used to describe latent class prevalences) and M*K item thresholds (i.e., **t**; measurement parameters used to describe item response probabilities). Thus, in a typical unrestricted LCA with binary indicators, *P* equals (*K-1*) + (M*K). In a typical (i.e., non-hierarchical) set of binary indicators, *W* equals 2^* M*^*.* Thus, a necessary condition for LCA identification is *W*–*P*-1 ≥ 0*.*

Recall that *W* in each hierarchical PASE Scale (where **u** = *u*_1_, *u*_2_,…*u*_6_) equaled 7 (or *M* + 1; see Table 2) by design, which was far less than 2^* M*^ (i.e., 64). For this reason, we used separate notation to distinguish between the number of possible response patterns in a typical set of binary indicators (i.e., *W* = 2^* M*^) from the number of possible response patterns in each PASE Scale (i.e., W* = *M* + 1). Thus, the maximum value of *P* in an identified LCA for each PASE Scale was 6 (i.e., 7-*P*-1 ≥ 0). If an unrestricted LCA was specified in each PASE Scale, such as when *K* = 2 and *P* = 13 (e.g., a_1_, t_11_, t_21_, t_31_, t_41_, t_51_, t_61_, t_12_, t_22_, t_32_, t_42_, t_52_, t_62_), *P* would quickly exceed W*—1 leaving the model unidentified.

One solution to this under identification was to restrict parameter estimation a priori via **t** based on the hierarchical structure of each PASE Scale (e.g., Guttman, 1944; Rindskopf, 1983). Specifically, elements in **t** (i.e., t_jk_) could be fixed to match the item response patterns depicted in Table 2, where *K* = 7, and 6 undefined elements in **a** (e.g., a_1_, a_2_, a_3_, a_4_, a_5_, a_6_) could be specified for estimation (i.e., *P* = 6). For example, on the inverse logit scale (Muthén, 1998–2004), the first six **t** elements (i.e., t_j1_) could each be fixed to 15 (i.e., ~ 0 probability of a yes response) to match the < 10 min item response pattern (i.e., 0,0,0,0,0,0); the next six **t** elements (i.e., t_j2_) could each be fixed to -15 (i.e., ~ 1 probability of a yes response), 15, 15, 15, 15, 15, respectively, to match the 10 min item response pattern (i.e., 1,0,0,0,0,0); etc. This approach was not pursued because we deemed it more confirmatory than exploratory, more observed than latent regarding the classes, and not very parsimonious (e.g., including classes with low prevalences such as the 120 min level).

A more exploratory approach was used to determine *K* for each PASE Scale. This approach relied on an algorithm implemented in M*plus* that automatically (i.e., does not require special code by the user) fixed elements in **t** that approached a boundary (i.e., a probability of 0 or 1) and/or **a** (i.e., to avoid singularity of the information matrix) to stabilize estimation (Muthén, 1998–2004). This algorithm was used from this point forward. This approach led us to use separate notation to distinguish between the number of parameters initially attempted to be estimated at the model specification stage (i.e., *P*) versus the number of parameters estimated in the converged ML solution (P*). Our expectation was that *P* would equal (*K-1*) + (M*K), while P* would equal ≤ 6, and thus, *P* > P* when *K* ≥ 2.

The number of latent classes accepted to explain response patterns to each PASE Scale was driven by empirical evidence (e.g., quality indexes for each solution) and substantive theory. Empirical evidence included a set of indicators commonly used in LCA (e.g., Collins & Lanza, 2010; Masyn, 2013; Morgan, 2015; Nylund et al., 2007): likelihood-ratio statistic (*G*^2^; Agresti, 1990), Akaike information criterion (AIC; Akaike, 1974), Bayesian information criterion (BIC; Schwarz, 1978), adjusted BIC (aBIC; Sclove, 1987), and the log of the likelihood function value at the ML solution (*LL*). We modified some of these indicators due to differences between *P* and P* and *W* and W*. For example, for AIC, BIC, and aBIC, we replaced *P* in each computation (i.e., AIC = *G*^2^ + *P*; BIC = *G*^2^ + [log(*N*)]*P*; aBIC = *G*^2^ + [log((*N* + 2)/*24*)]*P*) with P*. Similarly, although we considered the value of *G*^2^ we did not assume that this statistic followed the central chi-square distribution with *df* = *W* – *P – 1*. Extraction of *K* stopped if *G*^2^ = 0 because this indicated that the frequency of observed and expected response patterns were identical. Entropy (e.g., Ramaswamy et al., 1993) and the percentage of times a solution was selected out of 100 random sets of starting values (% Converged) also were provided for descriptive purposes.

Substantive evidence included the conceptual interpretability of estimated model parameters. Item thresholds with high homogeneity (i.e., degree to which item response probabilities for a particular latent class approached a boundary value) and separation (i.e., latent classes distinguished from each other) were desirable. Latent class prevalence estimates (based on estimated posterior probabilities) that matched substantive expectations based on measurement of self-efficacy level constructs in general (e.g., a class at or near the floor, ~ 10 min, and a class at or near the ceiling, ~ 150 min) and physical activity self-efficacy strength in adults with obesity in particular (e.g., an ~ up to 60 min class and a ~ 60 min or more class) were preferred. The arbitrary ordering of estimated classes in the output was re-ordered as needed (i.e., from lowest to highest physical activity self-efficacy level) from this point forward to make the solution more conceptually interpretable (e.g., Collins & Lanza, 2010).

### Results

The LCA model fit the data better as *K* increased from 1 to 4 in each PASE Scale at baseline (see Table 3). The number of parameters estimated in the converged solution generally stayed at its proposed maximum (i.e., P* = 6) as *K* increased. Extraction of *K* stopped at 4 because *G*^2^ = 0. A similar pattern of model-data fit supporting *K* = 4 was observed at 30 days post-baseline in each PASE Scale (see Table 1s in the supplemental materials). Note when *K* = 4 and a single element in **t** was estimated in each class then the maximum number of classes was four assuming no further restrictions on **a**.

**Table 3:** Number of latent classes to explain response patterns to each physical activity self-efficacy (PASE) scale at baseline

PASE Scale	Number of Latent Classes	P	P*	G2	AIC	BIC	aBIC	Entropy	% Converged	LL
Work-related moderate intensity (WRMI)	1	6	6	1821	3469	3494	3475	–	100	− 1728.74
	2	13	6	377	2025	2050	2030	1.00	98	− 1006.36
	3	20	6	43	1692	1716	1697	1.00	99	− 839.76
	**4**	**27**	**6**	**0**	**1648**	**1673**	**1654**	**0.99**	**83**	− **818.13**
Transport-related moderate intensity (TRMI)	1	6	6	1609	3302	3327	3308	–	100	− 1644.94
	2	13	6	374	2067	2091	2072	1.00	88	− 1027.34
	3	20	6	46	1738	1763	1744	1.00	77	− 863.24
	**4**	**27**	**6**	**0**	**1692**	**1717**	**1698**	**0.92**	**81**	− **840.23**
Domestic-related moderate intensity (DRMI)	1	6	6	1569	3220	3245	3226	–	100	− 1604.09
	2	13	6	350	2001	2026	2007	1.00	53	− 994.57
	3	20	6	47	1698	1723	1704	1.00	60	− 843.23
	**4**	**27**	**6**	**0**	**1651**	**1676**	**1657**	**0.96**	**77**	− **819.65**
Leisure-related moderate intensity (LRMI)	1	6	6	1574	3229	3254	3235	–	100	− 1608.73
	2	13	6	348	2004	2028	2009	1.00	44	− 995.84
	3	20	6	45	1701	1725	1706	1.00	67	− 844.25
	**4**	**27**	**5**	**0**	**1653**	**1674**	**1658**	**1.00**	**77**	− **821.61**

N = 461. Selected latent class model is in bold to facilitate interpretation. P = number of parameters initially attempted to be estimated at model specification; P* = number of parameters estimated in the converged solution; G 2 = likelihood-ratio statistic; AIC = Akaike information criterion; BIC = Bayesian information criterion; aBIC = sample-size adjusted Bayesian information criterion; % Converged = percentage of times a solution was selected out of 100 random sets of starting values;

LL = log of likelihood function value

Parameter estimates from the *K* = 4 solution for each PASE Scale were conceptually interpretable at baseline (see Table 4). In each class, no more than one element in **t** was estimated (i.e., at least 5 of the 6 relevant elements in **t** were fixed to a boundary value). Item response probabilities were homogeneous prior to (i.e., fixed to a probability of 1), and after (i.e., fixed to a probability of 0), $\hat{\tau }$ in each class. Classes were separated from each other (e.g., the physical activity self-efficacy level implied increased as class number increased). This separation allowed us to give each latent class a unique name based on the estimated item-response probability in the given column: 0.01–0.29 = probably the prior level; 0.30–0.69 = maybe the observed level; 0.70–0.99 = probably the observed level. For example, in the WRMI PASE Scale the four classes were labeled: Maybe 10 min ($\hat{\tau }_{11} =.41$), Maybe 60 min ($\hat{\tau }_{32} =.46$), Probably 90 min ($\hat{\tau }_{43} =.97$), and Probably 150 min ($\hat{\tau }_{64} =.92$). A similar set of class names/levels was assigned at baseline (i.e., ~ 10 min, ~ 60 min, ~ 90 min, and ~ 150 min) in each PASE Scale. Latent class prevalences also were similar across PASE Scales ranging from: 0.22–0.34 in the ~ 10 min class, 0.23–0.34 in the ~ 60 min class, 0.08 to 0.18 in the ~ 90 min class, and 0.30–0.34 in the ~ 150 min class.

**Table 4:** Parameter estimates from the four-class latent class model for each physical activity self-efficacy scale at baseline

Work-related moderate intensity (WRMI)				
Latent Class Name^a^	Maybe 10 min	Maybe 60 min	Probably 90 min	Probably 150 min
Latent Class Probabilities and (Counts)	0.34 (156)	0.26 (119)	0.08 (35)	0.33 (151)
Item-Response Probabilities^b^				
10 min	0.41***	**1.00**	**1.00**	**1.00**
30 min	0.00	**1.00**	**1.00**	**1.00**
60 min	0.00	0.46***	**1.00**	**1.00**
90 min	0.00	0.00	0.97*	**1.00**
120 min	0.00	0.00	0.00	**1.00**
150 min	0.00	0.00	0.00	0.92***

Transport-related moderate intensity (TRMI)				
Latent Class Name	Maybe 10 min	Maybe 60 min	Maybe 90 min	Probably 150 min
Latent Class Probabilities and (Counts)	0.30 (136)	0.23 (106)	0.18 (82)	0.30 (137)
Item-Response Probabilities				
10 min	0.50***	**1.00**	**1.00**	**1.00**
30 min	0.00	**1.00**	**1.00**	**1.00**
60 min	0.00	0.31***	**1.00**	**1.00**
90 min	0.00	0.00	0.49***	**1.00**
120 min	0.00	0.00	0.00	**1.00**
150 min	0.00	0.00	0.00	0.90***

Domestic-related moderate intensity (DRMI)				
Latent Class Name	Maybe 10 min	Maybe 60 min	Probably 90 min	Probably 150 min
Latent Class Probabilities and (Counts)	0.23 (105)	0.32 (146)	0.11 (52)	0.34 (158)
Item-Response Probabilities				
10 min	0.56***	**1.00**	**1.00**	**1.00**
30 min	0.00	**1.00**	**1.00**	**1.00**
60 min	0.00	0.49***	**1.00**	**1.00**
90 min	0.00	0.00	0.82***	**1.00**
120 min	0.00	0.00	0.00	**1.00**
150 min	0.00	0.00	0.00	0.92***

Leisure-related moderate intensity (LRMI)				
Latent Class Name	Maybe 10 min	Maybe 60 min	Probably 90 min	Above 150 min
Latent Class Probabilities and (Counts)	0.22 (102)	0.34 (155)	0.12 (57)	0.32 (147)
Item-Response Probabilities				
10 min	0.53***	**1.00**	**1.00**	**1.00**
30 min	0.00	**1.00**	**1.00**	**1.00**
60 min	0.00	0.52***	**1.00**	**1.00**
90 min	0.00	0.00	**1.00**	**1.00**
120 min	0.00	0.00	0.28***	**1.00**
150 min	0.00	0.00	0.00	**1.00**

*N* = 461. Non-zero item-response probabilities are in bold to facilitate interpretation

^a^Each latent class name was created based on the estimated non-zero item-response probability in the given column: .01-.29 = probably the prior level; .30-.69 = maybe the observed level; .70-.99 = probably the observed level

^b^Probabilities approaching a boundary (i.e., 0 or 1) were fixed by Mplus to stabilize estimation

^*^p < 0.05. ***p < 0.001

A similar pattern of parameter estimates was observed at 30 days post-baseline in each PASE Scale (see Table 5). While the pattern of parameter estimates appeared to be similar at 30 days post-baseline and baseline, a few noticeable differences were informally observed. For example, the second WRMI PASE Scale class was at a shorter duration level at 30 days post baseline (e.g., 30 min) than at baseline (e.g., 60 min). A pattern of a shorter duration level for the second class at 30 days post baseline than at baseline was observed across the PASE Scales. Whether this difference in the duration level of the second class across the paired PASE Scales by time reached the threshold of temporal measurement non-invariance, particularly given the constant duration in the first (e.g., 10 min), and the fourth (e.g., 150 min) class in each paired PASE Scale by time, was a question to be formally investigated in Study 2.

**Table 5:** Parameter Estimates from the Four-Class Latent Class Model for each Physical ActivitySelf-Efficacy Scale at 30 Days Post-Baseline

Work-related moderate intensity (WRMI)^a^				
Latent Class Name^b^	Below 10 min	Probably 30 min	Maybe 90 min	Probably 150 min
Latent Class Probabilities and (Counts)	0.26 (110)	0.26 (111)	0.25 (107)	0.23 (100)
Item-Response Probabilities^c^				
10 min	**0.22**	**1.00**	**1.00**	**1.00**
30 min	0.00	0.74***	**1.00**	**1.00**
60 min	0.00	0.00	**1.00**	**1.00**
90 min	0.00	0.00	0.39*	**1.00**
120 min	0.00	0.00	0.00	**1.00**
150 min	0.00	0.00	0.00	0.90***

Transport-related moderate intensity (TRMI)				
Latent Class Name	Maybe 10 min	Probably 30 min	Maybe 90 min	Probably 150 min
Latent Class Probabilities and (Counts)	0.33 (142)	0.25 (107)	0.19 (79)	0.23 (99)
Item-Response Probabilities				
10 min	0.42***	**1.00**	**1.00**	**1.00**
30 min	0.00	**1.00**	**1.00**	**1.00**
60 min	0.00	0.20*	**1.00**	**1.00**
90 min	0.00	0.00	0.58***	**1.00**
120 min	0.00	0.00	0.00	**1.00**
150 min	0.00	0.00	0.00	0.83***

Domestic-related moderate intensity (DRMI)				
Latent Class Name	Below 10 min	Probably 30 min	Maybe 90 min	Probably 150 min
Latent Class Probabilities and (Counts)	.12 (51)	.35 (149)	.26 (113)	.27 (114)
Item-Response Probabilities				
10 min	**0.06**	**1.00**	**1.00**	**1.00**
30 min	0.00	0.72***	**1.00**	**1.00**
60 min	0.00	0.00	**1.00**	**1.00**
90 min	0.00	0.00	0.42***	**1.00**
120 min	0.00	0.00	0.00	**1.00**
150 min	0.00	0.00	0.00	0.90***

Leisure-related moderate intensity (LRMI)				
Latent Class Name	Maybe 10 min	Probably 10 min	Maybe 60 min	Probably 150 min
Latent Class Probabilities and (Counts)	0.24 (101)	0.12 (50)	0.37 (159)	0.27 (117)
Item-Response Probabilities				
10 min	0.47***	0.98***	**1.00**	**1.00**
30 min	0.00	0.00	**1.00**	**1.00**
60 min	0.00	0.00	0.46***	**1.00**
90 min	0.00	0.00	0.00	**1.00**
120 min	0.00	0.00	0.00	**1.00**
150 min	0.00	0.00	0.00	0.86***

*N* = 427. Non-zero item-response probabilities are in bold to facilitate interpretation

^a^*N* = 428

^b^Each latent class name was created based on the estimated non-zero item-response probability in the given column: 0.01–0.29 = probably the prior level; 0.30–0.69 = maybe the observed level; 0.70–0.99 = probably the observed level

^c^Probabilities approaching a boundary (i.e., 0 or 1) were fixed by Mplus to stabilize estimation

*p < 0.05. ***p < 0.001

### Conclusion

A reasonable answer to the first research question was that four classes explained response patterns to each PASE Scale at baseline and 30 days post-baseline independently. This person-centered conclusion (e.g., four discreet classes at baseline: ~ 10 min, ~ 30 min or ~ 60 min, ~ 90 min, and ~ 150 min) is different than the variable-centered conclusion (i.e., two continuous factors: up to 60 min and 60 min or more) put forth by Myers et al. (2021a, 2021b) and may have implications for findings regarding the effectiveness of FFW to promote physical activity self-efficacy strength (as in Myers, Bateman et al.) versus level (focus of the current study). Prior to investigating this possibility in Study 4, however, we first investigated evidence for temporal measurement invariance of this four-class solution in Study 2. Providing evidence for temporal measurement invariance of a LCA is necessary to establish that classes are comparable (e.g., group differences in latent class prevalences) across time (e.g., Millsap, 2012).

## Study 2: temporal measurement invariance

### Method

Measurement invariance by time was evaluated by fitting two nested LCA models (e.g., Collins & Lanza, 2010; Masyn, 2017) in each PASE Scale. Model 1 estimated *K* = 4 at baseline (i.e., *C*_1_) and 30 days post-baseline (i.e., *C*_2_) simultaneously with no parameter restrictions by *C* at model specification. Model 2 added the constraint of invariant ${{\boldsymbol{\uptau}}}$ by *C* to Model 1. The fit of these two models was evaluated with a subset of the empirical indicators used in Study 1 (e.g., *G*^2^, AIC, BIC, and aBIC) and the likelihood ratio difference test (LRT): -2(*LL*_Model 1_—*LL*_Model 2_) with *df* = P*_Model 1_—P*_Model 2_. Conceptual interpretability of estimated model parameters was evaluated as in Study 1 (i.e., homogeneity; separation; prevalences; substantive theory).

### Results

Table 6 provides key results of latent class measurement invariance by time analyses for each PASE Scale. Model 2 (i.e., invariant **t**) was accepted in each PASE Scale because (a) this model did not fit the data significantly worse than the less restricted model (i.e., noninvariant **t**) and (b) its parameter estimates were conceptually interpretable. For example, a similar (compared to Study 1) set of unique class names/levels was assigned (i.e., ~ 10 min, ~ 30 min or ~ 60 min, ~ 90 min, and ~ 150 min) in each PASE Scale across time. Latent class prevalences, which were free to vary by time, were similar across PASE Scales at both time points. At baseline prevalences ranged from: 0.22–0.34 in the ~ 10 min class, 0.16–0.34 in the ~ 30 min or ~ 60 min class, 0.08–0.27 in the ~ 90 min class, and 0.27–0.34 in the ~ 150 min class. Similarly, at 30 days post-baseline prevalences ranged from: 0.22–0.33 in the ~ 10 min class, 0.25–0.37 in the ~ 30 min or ~ 60 min class, 0.11–0.26 in the ~ 90 min class, and 0.19–0.27 in the ~ 150 min class. Table 2s in the supplemental materials provides parameter estimates from Model 2 in each PASE Scale.

**Table 6:** Latent Class Measurement Invariance by Time (i.e., Baseline and 30 Days Post-Baseline) for each Physical Activity Self-Efficacy (PASE) Scale

PASE Scale	Model	Par	Par*	G2	AIC	BIC	aBIC	LL	LRT^a^
Work-related moderate intensity (WRMI)	Noninvariant	54	12	275.06	3225	3274	3236	− 1600.26	0.42(2) *p* = 0.811
	Invariant	30	10	275.48	3221	3262	3231	− 1600.47	
Transport-related moderate intensity (TRMI)	Noninvariant	54	12	267.97	3295	3345	3306	− 1635.48	5.80(2) *p* = 0.055
	Invariant	30	10	273.77	3297	3338	3306	− 1638.37	
Domestic-related moderate intensity (DRMI)	Noninvariant	54	13	273.60	3205	3259	3218	− 1589.75	2.82(4) *p* = 0.588
	Invariant	30	9	276.42	3200	3238	3209	− 1591.16	
Leisure-related moderate intensity (LRMI)	Noninvariant	54	12	295.12	3246	3295	3257	− 1610.94	2.10(2) *p* = 0.350
	Invariant	30	10	297.22	3244	3285	3254	− 1611.99	

N = 461. Selected latent class model is in bold to facilitate interpretation. Par = number of parameters initially attempted to be estimated by Mplus; Par* = number of parameters estimated by Mplus; G 2 = likelihood-ratio statistic; AIC = Akaike information criterion; BIC = Bayesian information criterion; aBIC = sample-size adjusted Bayesian information criterion; LL = Log of likelihood function value; LRT = likelihood-ratio test

^a^Difference in df for the LRT (i.e., 4 versus 2) was due to was due to an algorithm implemented in Mplus that automatically fixed elements in tau that approached a boundary (i.e., a probability of 0 or 1) and/or alpha (i.e., to avoid singularity of the information matrix) to stabilize estimation and not due to the allowance of partial temporal measurement invariance

### Conclusion

A reasonable answer to the second research question was that there was strong evidence for temporal measurement invariance of the four-class LCA solution for each PASE Scale. This finding established that the meaning of latent classes was comparable at baseline (e.g., prior to intervention) and 30 days post-baseline (i.e., at the conclusion of intervention). Given this apparent comparability of classes across time, unconditional transition probabilities (i.e., stability and change in the four classes from baseline to 30 days post-baseline) were explored in Study 3.

Prior to beginning Study 3 latent class measurement invariance analyses by randomization group (i.e., UC versus FFW) were performed at both baseline and 30 days post-baseline. These analyses did not an answer a research question per se but did test an implicit assumption of latent class measurement invariance by randomization group of the four-class LCA solution for each PASE Scale at both timepoints. Table 3s and Table 4s in the supplemental materials provides key results of latent class measurement invariance by randomization group for each PASE Scale at both baseline and 30 days post-baseline, respectively. Model 2 (i.e., invariant **t**) was accepted in each PASE Scale because this model did not fit the data significantly worse than the less restricted model (i.e., noninvariant **t**) at both timepoints. Thus, evidence was provided for the assumption that the latent classes were comparable across randomization groups at both timepoints.

## Study 3: unconditional transitional probabilities

### Method

Unconditional transition probabilities were estimated via LTA prior to estimating conditional transitional probabilities (e.g., Collins & Lanza, 2010; Hagenaars & McCutcheon, 2002; Nylund-Gibson et al., 2023). Specifically, a four-class latent variable at 30 days post-baseline (i.e., *C*_2_) was regressed (in a multinomial logistic regression) on a four-class latent variable at baseline (i.e., *C*_1_) with temporal measurement invariance imposed in each PASE Scale. This matrix of direct effects (**B**) and **a** yielded a four-by-four transition probability matrix (**P**). Estimates on the (main) diagonal in **P** indicated stability. For example, $\hat{p}_{11}$ estimated the probability of membership in class 1 at 30 days post-baseline given membership in class 1 at baseline. Estimates off the diagonal in **P** indicated change. For example, $\hat{p}_{12}$ estimated the probability of membership in class 2 at 30 days post-baseline given membership in class 1 at baseline. Because classes were ordered from lowest to highest, elements to the right of the diagonal in **P** indicated an increase in physical activity self-efficacy level (e.g., moving up from the ~ 10 min class to the ~ 60 min class) from baseline to 30 days post-baseline. Conversely, elements to the left of the diagonal in **P** indicated a decrease in physical activity self-efficacy level (e.g., moving back from the ~ 60 min class to the ~ 10 min class) from baseline to 30 days post-baseline. Statistical significance of elements in ${\hat{\mathbf{P}}}$ was evaluated with the model constraint command (see Appendix F in the supplemental materials for an example of M*plus* input code).

### Results

Table 7 provides ${\hat{\mathbf{P}}}$ for each PASE Scale. Estimates on the diagonal hovered around 0.50 (i.e., ranged from 0.38 to 0.67) and were statistically significant in each PASE Scale. These values indicated that the probability of remaining in the same class at both baseline and 30 days post-baseline was around 0.50, but also suggested an approximately equal probability of switching classes from baseline to 30 days post-baseline. Estimates off the diagonal varied (i.e., ranged from 0.02 to 0.50) and typically were statistically significant in each PASE Scale. These values indicated that the probability of switching to a particular class from baseline to 30 days post-baseline (i.e., change) was typically greater than zero – with the largest change value in each row generally adjacent to the diagonal (i.e., a one class increase or decrease in physical activity self-efficacy level). For example, in the WRMI PASE Scale, the probability of moving up from the ~ 10 min class at baseline to the ~ 60 min class at 30 days post-baseline was 0.40 whereas the probability of moving up to the ~ 90 min class/ ~ 150 min class was 0.03/0.05, respectively.

**Table 7:** Unconditional transition probabilities at 30 days post-baseline by latent classes at baseline for each physical activity self-efficacy scale

Work-related moderate intensity (WRMI)				
Latent Class Name	Maybe 10 min at 30 Days	Maybe 60 min at 30 Days	Probably 90 min at 30 Days	Probably 150 min at 30 Days
Maybe 10 min at Baseline	0.52***	0.40**	0.03	0.05**
Maybe 60 min at Baseline	0.45***	0.47***	0.05	0.03
Probably 90 min at Baseline	0.12*	0.23**	0.52***	0.13*
Probably 150 min at Baseline	0.12***	0.07**	0.20***	0.61***

Transport-related moderate intensity (TRMI)				
Latent Class Name	Below 10 min at 30 Days	Maybe 30 min at 30 Days	Maybe 90 min at 30 Days	Probably 150 min at 30 Days
Below 10 min at Baseline	0.48***	0.20***	0.19***	0.13**
Maybe 30 min at Baseline	0.19***	0.67***	0.10***	0.04*
Maybe 90 min at Baseline	0.16***	0.25***	0.42***	0.16***
Probably 150 min at Baseline	0.08**	0.11***	0.24***	0.57***

Domestic-related moderate intensity (DRMI)				
Latent Class Name	Maybe 10 min at 30 Days	Probably 30 min at 30 Days	Maybe 90 min at 30 Days	Probably 150 min at 30 Days
Maybe 10 min at Baseline	0.43***	0.50***	0.02	0.05*
Probably 30 min at Baseline	0.28***	0.57***	0.09	0.06*
Maybe 90 min at Baseline	0.17***	0.23***	0.44***	0.16***
Probably 150 min at Baseline	0.06**	0.09**	0.21***	0.64***

Leisure-related moderate intensity (LRMI)				
Latent Class Name	Below 10 min at 30 Days	Maybe 30 min at 30 Days	Maybe 90 min at 30 Days	Probably 150 min at 30 Days
Below 10 min at Baseline	0.38***	0.27***	0.20**	0.16**
Maybe 30 min at Baseline	0.08**	0.67***	0.21***	0.04*
Maybe 90 min at Baseline	0.17***	0.23***	0.48***	0.12***
Probably 150 min at Baseline	0.05**	0.09***	0.22***	0.65***

*N* = 461. Transition probabilities estimating stability are in bold to facilitate interpretation

*p < 0.05. **p < 0.01. ***p < 0.001

### Conclusion

There were two reasonable and related answers to the third research question. The first answer was that there was modest stability in class membership from baseline (i.e., prior to intervention) to 30 days post-baseline (i.e., at the conclusion of intervention) in each PASE Scale. The second answer was that specific changes in class membership from baseline to 30 days post-baseline varied (i.e., substantial increases and decreases in physical activity self-efficacy level were observed) in each PASE Scale. Given this level and dispersion of change in class membership from baseline to 30 days post-baseline, and the design of the WBPA study (i.e., assignment to the FFW group was intended to increase physical activity self-efficacy), conditional transition probabilities by intervention group were explored in Study 4.

## Study 4: conditional transitional probabilities by intervention group

### Method

Conditional transition probabilities by intervention group were estimated via a multiple-group LTA (e.g., Collins & Lanza, 2010; Hagenaars & McCutcheon, 2002; Nylund-Gibson et al., 2023). Observed groups (i.e., UC = 0, FFW = 1) were defined with the knownclass (i.e., *C*_g_) command in M*plus*. The model was specified as in Study 3 with one exception: **B** was to be estimated separately in each intervention group (i.e., ${\mathbf{\rm B}}_{{{\mathrm{UC}}}},{\mathbf{\rm B}}_{{{\mathrm{FFW}}}}$). This difference in model specification allowed **P** to vary by intervention group (i.e., ${\mathbf{P}}_{{{\mathrm{UC}}}},{\mathbf{P}}_{{{\mathrm{FFW}}}}$).

Differences in **P** (i.e., ${\mathbf{P}}_{{{\mathrm{FFW}}}} - {\mathbf{P}}_{{{\mathrm{UC}}}} = {\mathbf{P}}_{{{\mathrm{difference}}}}$) were the focal parameters (e.g., Clogg & Goodman, 1985; Schmiege et al., 2018). Elements on the diagonal in **P**_difference_ indicated differences in stability by intervention group. For example,$p_{{11\_{\mathrm{difference}}}}$ indicated the difference in probability of membership in class 1 at 30 days post-baseline given membership in class 1 at baseline in the FFW group versus the UC group. Elements off the diagonal in **P**_difference_ indicated differences in change by intervention group. For example, $p_{{{\mathrm{12\_difference}}}}$ indicated the difference in probability of membership in class 2 at 30 days post-baseline given membership in class 1 at baseline in the FFW group versus the UC group. Because classes were ordered from lowest to highest, positively signed elements to the right of the diagonal in **P**_difference_ indicated more of an increase in physical activity self-efficacy level (e.g., moving up from the ~ 10 min class to the ~ 60 min class) from baseline to 30 days post-baseline– in the FFW group versus the UC group. Conversely, negatively signed elements to the left of the diagonal in **P** indicated less of a decrease in physical activity self-efficacy level (e.g., moving down from the ~ 60 min class to the ~ 10 min class) from baseline to 30 days post-baseline in the FFW group versus the UC group. Statistical significance of elements in ${\mathbf{P}}_{{{\mathrm{difference}}}}$ was evaluated with the model constraint command (see Appendix G in the supplemental materials for an example of M*plus* input code).

### Results

Table 8 provides ${\hat{\mathbf{P}}}_{{{\mathrm{difference}}}}$ for each PASE Scale. Most estimates within ${\hat{\mathbf{P}}}_{{{\mathrm{difference}}}}$ were statistically non-significant—though at least one element was statistically significant—in each PASE Scale. Moreover, there was a clear trend in the statistically significant elements in ${\hat{\mathbf{P}}}_{{{\mathrm{difference}}}}$ across PASE Scales. Specifically, $\hat{p}_{{11\_{\mathrm{difference}}}}$ was statistically significant and negative in each PASE Scale. This meant that the probability of remaining in the lowest physical activity self-efficacy level (i.e., ~ 10 min class) at 30 days post-baseline (given membership in this lowest class at baseline) was smaller in the FFW group (i.e., less stable) versus the UC group. For example, in the WRMI PASE Scale, $\hat{p}_{{11\_{\mathrm{difference}}}} = -.18$ because $\hat{p}_{{{\mathrm{11\_FFW}}}} =.43$ while $\hat{p}_{{{\mathrm{11\_UC}}}} =.61$. In most PASE Scales, an off-diagonal element in this first row was statistically significant and positive, which indicated more movement from the lowest physical activity self-efficacy level to a specific higher physical activity self-efficacy level – in the FFW group (i.e., more positive change) versus the UC group. For example, in the WRMI PASE Scale, $\hat{p}_{{14\_{\mathrm{difference}}}} =.09$ because $\hat{p}_{{{\mathrm{14\_FFW}}}} =.10$ while $\hat{p}_{{{\mathrm{14\_UC}}}} =.01$. This meant that the probability of moving from the lowest WRMI PASE class at baseline (i.e., ~ 10 min class) to the highest WRMI PASE class at 30 days post-baseline (i.e., ~ 150 min class) was greater for FFW group versus the UC group (i.e., more positive change). The only statistically significant element in a ${\hat{\mathbf{P}}}_{{{\mathrm{difference}}}}$ that was not in the first row was $\hat{p}_{{21\_{\mathrm{difference}}}} = -.11$ in the DRMI PASE Scale. The meaning of this element was somewhat different (i.e., less moving back from the ~ 30 min class at baseline to the ~ 10 min class at 30 days post-baseline), which indicated less negative change for the FFW group (i.e.,$\hat{p}_{{{\mathrm{21\_FFW}}}} =.04$) versus the UC group (i.e., $\hat{p}_{{{\mathrm{21\_UC}}}} =.15$). Table 5s in the supplemental materials provides ${\hat{\mathbf{P}}}_{{{\mathrm{FFW}}}}$ and ${\hat{\mathbf{P}}}_{{{\mathrm{UC}}}}$ from this LTA in each PASE Scale.

**Table 8:** Difference in transition probabilities at 30 days post-baseline by latent classes at baseline for the fun for wellness group versus the usual care group for each physical activity self-efficacy scale

Work-related moderate intensity (WRMI)				
Latent Class Name	Maybe 10 min at 30 Days	Maybe 60 min at 30 Days	Probably 90 min at 30 Days	Probably 150 min at 30 Days
Maybe 10 min at Baseline	− 0.18*	0.14	− 0.05	0.09*
Maybe 60 min at Baseline	− 0.08	0.18	− 0.04	− 0.06
Probably 90 min at Baseline	0.05	− 0.22	0.21	− 0.04
Probably 150 min at Baseline	0.00	0.01	− 0.01	0.01

Transport-related moderate intensity (TRMI)				
Latent Class Name	Below 10 min at 30 Days	Maybe 30 min at 30 Days	Maybe 90 min at 30 Days	Probably 150 min at 30 Days
Below 10 min at Baseline	− 0.33**	0.10	0.06	0.17
Maybe 30 min at Baseline	− 0.11	0.03	0.07	0.01
Maybe 90 min at Baseline	− 0.02	0.00	0.07	− 0.05
Probably 150 min at Baseline	0.03	0.07	− 0.11	0.02

Domestic-related moderate intensity (DRMI)				
Latent Class Name	Below 10 min at 30 Days	Maybe 30 min at 30 Days	Maybe 90 min at 30 Days	Probably 150 min at 30 Days
Below 10 min at Baseline	− 0.36**	− 0.08	0.34**	0.10
Maybe 30 min at Baseline	− 0.11*	0.00	0.08	0.03
Maybe 90 min at Baseline	− 0.08	− 0.06	0.11	0.02
Probably 150 min at Baseline	0.04	0.02	0.01	− 0.07

Leisure-related moderate intensity (LRMI)				
Latent Class Name	Below 10 min at 30 Days	Maybe 30 min at 30 Days	Maybe 90 min at 30 Days	Probably 150 min at 30 Days
Below 10 min at Baseline	− 0.32*	− 0.05	0.34**	0.03
Maybe 30 min at Baseline	− 0.07	0.06	0.05	− 0.05
Maybe 90 min at Baseline	− 0.01	− 0.03	0.01	0.02
Probably 150 min at Baseline	0.05	0.04	0.02	− 0.11

*N* = 461. Statistically significant differences in transition probabilities are in bold bold to facilitate interpretation

*p < 0.05. **p < 0.01

### Post Hoc* LTA with covariates*

#### Method

To check the stability of **P**_difference_ a set of possible covariates of physical activity self-efficacy collected at baseline were specified as simultaneous predictors of *C*_2_ within the multiple-group LTA already described (Nylund-Gibson et al., 2023). This set of possible covariates was based on theory and previous research (e.g., Bandura, 1977, 1997; Bauman et al., 2012; Rubenstein et al., 2016) and included: physical activity, gender, race/ethnicity, highest level of education completed, marital status, employment status, age, and household annual income. Each of these covariates was centered at its sample mean. The effect of each of these covariates on *C*_2_ was modeled as invariant by *C*_g_ (i.e., randomization group). Given that each of these covariates was collected at baseline, and that assignment to *C*_g_ also occurred at baseline, our expectation was that **P**_difference_ (at the sample mean for each covariate) would be relatively stable with the addition of these covariates to the LTA. Given that the focus of this post hoc LTA was on the stability of **P**_difference_ and not the regression coefficients from the covariates to *C*_2_, estimates of these regression coefficients were not explored unless the post hoc LTA model that included these covariates was retained.

#### Results

Table 6s in the supplemental materials provides ${\hat{\mathbf{P}}}_{{{\mathrm{difference}}}}$(at the sample mean for each covariate) in each PASE Scale. As can be viewed in Table 6s, ${\hat{\mathbf{P}}}_{{{\mathrm{difference}}}}$ was relatively stable (i.e., similar to Table 7) with the addition of a set of possible covariates of physical activity self-efficacy collected at baseline specified as predictors of *C*_2_. For ease of interpretation, we focus on the multiple-group LTA without this set of covariates from this point forward and refer readers to Muthén and Asparouhov (2011) for details on LTA with covariates.

### Conclusion

A reasonable answer to the fourth research question was that there was mixed evidence for transition probabilities differing by intervention group in each PASE Scale. This mixture of evidence seemed to depend on physical activity self-efficacy class at baseline. About one-half (i.e., 7/16) of the transition probability estimates from the lowest physical activity self-efficacy level (i.e., ~ 10 min class) at baseline to 30 days post-baseline differed by intervention group and in each case was a positive result for the FFW group compared to the UC group. Conversely, almost none of the transition probability estimates (i.e., 1/48) from the higher physical activity self-efficacy levels (i.e., ~ 30 min class and beyond) at baseline to 30 days post-baseline differed by intervention group. These person-centered findings regarding physical activity self-efficacy level are compared to the variable-centered findings regarding physical activity self-efficacy strength put forth by Myers et al. (2021a, 2021b) in the brief general discussion section.

## Brief general discussion

The objective of this study was to measure stability and change in response patterns to a set of hierarchical PASE Scales with LTA. To accomplish this objective a multiple-group LTA modeled binary responses to six ordered items within each PASE Scale to explore class enumeration, temporal measurement invariance of the accepted LCA solution, unconditional transition probabilities from baseline (i.e., pre-intervention) to 30 days post-baseline (i.e., post-intervention), and conditional transition probabilities by intervention group in the WBPA study. The person-centered results reported in the current study, which used mixture models, extend results previously reported based on a traditional variable-centered approach, which used the common factor model with a single class. This extension has potential implications for the design of future FFW trials as well as other physical activity-promoting interventions in this unique and at-risk population (USPSTF, 2018).

Modeling change in physical activity self-efficacy beliefs in physical activity-promoting interventions for adults with obesity typically takes a traditional variable-centered approach (e.g., Bateman et al., 2022). Myers et al., (2021a, 2021b) took such an approach in the WBPA Study and put forth two variable-centered conclusions regarding the effectiveness of the FFW intervention to promote positive change in physical activity self-efficacy strength at 30 days post-baseline. The first conclusion was that there was uniform evidence for the effectiveness of the FFW intervention to promote positive change in work-, transport-, domestic-, and leisure-related latent physical activity self-efficacy strength in an up to 60 min continuous latent factor. The second conclusion was that there was uniform evidence for the ineffectiveness of the FFW intervention to promote positive change in work-, transport-, domestic-, and leisure-related latent physical activity self-efficacy strength in a 60 min or more continuous latent factor. This variable-centered approach yielded important variable-level insights but did not address for whom the FFW intervention may be effective in promoting physical activity self-efficacy beliefs.

The current study extends the findings put forth by Myers et al. (2021a, 2021b) by taking an uncommonly used person-centered approach and puts forth two person-centered conclusions regarding the effectiveness of the FFW intervention to promote positive change in physical activity self-efficacy level. The first conclusion is that there is uniform evidence for the effectiveness of the FFW intervention to promote at least some positive change in work-, transport-, domestic-, and leisure-related latent physical activity self-efficacy level in the lowest ordered discreet latent factor (i.e., ~ 10 min class). The second conclusion is that there is nearly uniform evidence for the ineffectiveness of the FFW intervention to promote significant positive change in work-, transport-, domestic-, and leisure-related latent physical activity self-efficacy level beyond the lowest ordered discreet latent factor (i.e., ~ 30 or 60 min, ~ 90 min, ~ 150 min classes). Next generation FFW trials may consider the use of physical activity self-efficacy level at baseline as a potential selection criterion (e.g., a low level) for participant enrollment. This type of adaptation would align with “personalized prevention” that better targets intervention components to an individual’s specific pre-existing risk factors (e.g., August & Gewirtz, 2019; Collins et al., 2004; Howe & Leitjen, 2023). Beyond the FFW intervention, other physical activity-promoting interventions for this at-risk population may benefit from secondary person-centered analyses on RCTs, like those in the current study, that attempt to identify for whom these interventions may be effective to better optimize future delivery of these interventions (e.g., Collins & Kugler, 2018; USDHHS, 2018; USPSTF, 2018; WHO, 2018). Another future line of inquiry could be person-centered analyses of physical activity self-efficacy strength (building off the physical activity self-efficacy level focus in the current manuscript) in RCTs.

Multiple-group LTA where the grouping variable is randomization to an intervention within a RCT is relatively rare (e.g., Lanza & Rhoades, 2013)—even though it has the potential to answer an important research question: do transition probabilities for latent classes of a theory-driven malleable intervention target differ by intervention groups? Thus, the current study might reasonably be considered an innovative application of “regular” LTA. We use the expression regular to distinguish the LTA approach taken in the current study from a recent random intercepts LTA (RI-LTA) approach put forth by Muthén and Asparouhov (2022). The RI-LTA approach attempts to separate between subject (e.g., trait) variation from within-subject (e.g., state) latent class transitions over time and may outperform (e.g., model-data fit; parameter estimate precision; etc.) regular LTA in some circumstances (e.g., when the data generating model follows a RI-LTA; e.g., Tseng, 2024). Unfortunately, we encountered estimation difficulties when we tried to fit a RI-LTA (with a single continuous random intercept factor with pattern coefficients invariant by time) to the data in the current study. These estimation difficulties were typically observed in the latent class measurement model with many estimates within ${\hat{\mathbf{\tau }}}$ taking extreme values (i.e., >|15|). These difficulties may have been related to the hierarchical nature of the PASE Scales (i.e., only non-hierarchical scales were reported in Muthén & Asparouhov) and/or the sample size in the current study (which was at least somewhat smaller than the real-data and simulated samples in Muthén & Asparouhov). The previously mentioned algorithm (see Study 1) implemented in M*plus* that automatically fixed elements in **t** that approached a boundary (i.e., a probability of 0 or 1) and/or **a** (i.e., to avoid singularity of the information matrix) to stabilize estimation did not seem to be activated in our RI-LTA approach (e.g., many estimates within ${\hat{\mathbf{\tau }}}$ taking extreme values: >|15|). Future methodological research that builds off the seminal work in Muthén & Asparouhov and studies the performance of RI-LTA under an even wider variety of conditions is needed (Nylund-Gibson et al., 2023).

## Data Availability

The dataset analyzed during the current study are not publicly available due to the requirement of data sharing agreements with the IRB mentioned in the Ethics Approval section. De-identified data will be made available upon request and approval of the relevant IRB(s). Because of this limitation, a synthetic dataset (i.e., an imputed dataset that closely resembles the dataset analyzed in the current study) is available in Appendix H of the Supplemental Materials.

## Code availability

The supplemental materials contain key M*plus* code. All M*plus* code will be made available upon request.
